# A Review of the Genus *Miresa* Walker in China (Lepidoptera: Limacodidae)

**DOI:** 10.1673/031.011.0134

**Published:** 2011-03-24

**Authors:** Chun-Sheng Wu, Alexey V. Solovyev

**Affiliations:** ^1^ Key Laboratory of Zoological Systematics and Evolution, Institute of Zoology, Chinese Academy of Sciences, Beijing 100101, P.R. China; ^2^ Department of Zoology, Ulyanovsk State Pedagogical University, Ulyanovsk 432700, Russia

**Keywords:** *Miresa fangae* sp. n., *Miresa polargenta* sp. n., *Miresa burmensi*, *Miresa argentifera kwangtungensis* stat. n., *Miresa bracteata*, *Miresa bracteata* var. *orientis*, *Miresa fulgida*, *Miresa urga*

## Abstract

Eight species of the genus *Miresa* Walker are recognized in China including two new species, *M. fangae* Wu & Solovyev and *M. polargenta* Wu & Solovyev, described herein. *M. burmensis* Hering species is reported for the first time in China. The *M. argentifera kwangtungensis* Hering, 1931 taxon is raised to full specific status. The lectotypes are designated for the following 5 taxa: *M. bracteata* Butler, 1880 (♂, Natural History Museum, London); *M. fulgida* Wileman, 1910 (♂, Natural History Museum, London); *M. bracteata* var. *orientis* Strand, 1915 (♂, Rijksmuseum van Natuurlijke Historie, Leiden); *M. argentifera kwangtungensis* Hering, 1931 (♂, Zoologisches Museum der Humboldt Universität zu Berlin) and *M. urga* Hering, 1933 (♂, Zoologisches Museum der Humboldt Universität zu Berlin). The photographs of moths and their genitalia are given, a key to the Chinese species of the genus is provided, and the distributional maps are also given.

## Introduction

The genus *Miresa* was erected by Walker in 1855 and it included 5 species from the Indian region. The type species, *Nyssia albipuncta* Herrich-Schäffer [1854] 1850–1858, was designated subsequently by Moore (1882–3: 128). The genus is presently considered pantropical - occurring in Asia, Africa, and America (van Eecke 1925; Hering 1928, 1931, 1933; Dyar 1935; Epstein, Corrales 2004) and includes more than 30 species. The check lists of the genus were given more than 70 years ago (van Eecke 1925; Hering 1928, 1931, 1933; Dyar 1935). These lists were slightly changed later by transferring some species to other genera, but these investigations involved just a few species and were connected with local faunistic studies (e.g. Janse 1964). Therefore, the lists are debatable at present. The recent diagnosis of the genus was given by Holloway (1986) and concerned Asian species only, although the monophyly of *Miresa* worldwide remained not evident and not supported. The genus needs revising worldwide to determine if taxa presently in the genus belong elsewhere. Perhaps it ranges to South-East Asia only, and the Asian and American members may belong to other genera.

The main aim of this paper is to overview Chinese *Miresa* to observe morphology and biology, and make some notes to their distribution and nomenclature. The examination of Chinese *Miresa* is urgent due to their economical importance. The larvae are famous pests of various agricultural plants and attribute to the “nettle”-type (Godfray et al. 1987), bearing stinging spurs that can cause burning pain and itching as a result of their contact with a human skin. No outbreaks in China have been reported up to now, but the biological control of *Miresa* population size is recommended.

Up to now 5 species of the genus were recorded from China (Leech 1899; Hering 1931, 1933; Cai 1981, 1982; Inoue 1992; Fang 1997), but the list needed improvement and reexamination of Chinese fauna. Misidentifications made in previous investigations were found; 3 species are added to Chinese fauna, including 2 new to science and 1 newly recorded species.

## Materials and Methods

Material examined for this study was based on the insect collections of the Institute of Zoology, Chinese Academy of Sciences, Beijing, P. R. China (IZCAS). Material from the Museum Witt Munich, Germany (MWM), and collections of A. V. Solovyev (CSAV) were also examined for this study. The type specimens of all mentioned taxa examined are kept in the Natural History Museum, London, United Kingdom (NHM), Zoologisches Museum der Humboldt Universität zu Berlin, Germany (ZMHB), Nationaal Natuurhistorische Museum (“Naturalis”) (Leiden, Netherlands (formerly the Rijksmuseum van Natuurlijke Historie), (RMNH), and Museum National d'Histoire Naturelle, Paris, France (MNHN). The photographs of moths and their genitalia are given. Standard methods of dissection and mounting in Euparal described by Holloway et al. (1987).

### Systematics

***Miresa* Walker, 1855**
*Miresa* Walker, 1855, *List Specimens lepid. Insects Colin Br. Mus*. 5: 1103, 1123. Type species: *Nyssia albipuncta* Herrich-Schäffer, [1854] 1850–1858, by subsequent designation by Moore 1882–3: 128.
  *Nyssia* Herrich-Schäffer, [1854] 1850–1858, *Samml. aussereurop. Schmett*. **1** (1): wrapper, Taf. 37, Figures 178, 179, preocc. name (Duponchel, 1829 [Geometridae]). Type species: *Nyssia albipuncta* Herrich-Schäffer, [1854] 1850–1858, by subsequent designation by Fletcher, Nye 1982: 104.
  *Neomiresa* Butler, 1878, *Trans, ent. Soc. Lond*. **1878**: 74. Type species: *Nyssia argentata* Walker, 1855, by original designation.
  *Miresopsis* Matsumura, 1927, *J. Coll. Agric. Hokkaido Imp. Univ*. **19**: 86, 87. Type species: *Miresa bracteata* Butler, 1880, by original designation.

**Figures 1–10.**
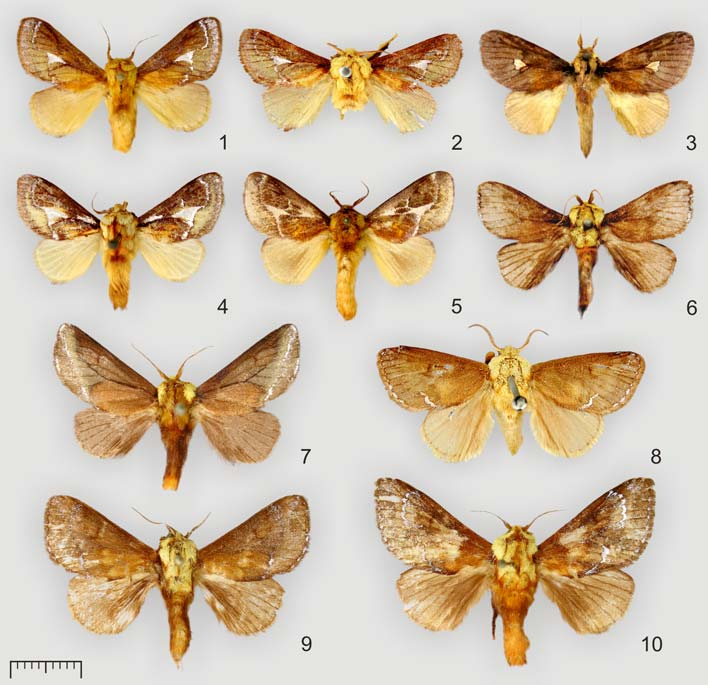
*Miresa* spp. **1.** *M. bracteata* Butler, ♂ (MWM); **2.** *M. bracteata* Butler, ♂, lectotype (NHM) [India]; **3.** *M. burmensis* Hering, ♂ (IZCAS); **4.** *M. demangei* de Joannis, ♂ (MWM); **5.** *M. fulgida* Wileman, ♂ (MWM); **6.** *M. kwangtungensis* Hering, ♂ (IZCAS); **7.** *M. urga* Hering, ♂ (MWM); **8.** *M. argentifera* Walker, ♂, holotype (NHM) [Ceylon]; **9.** *M. fangae* **sp. n.**, ♂, paratype, China Hainan (MWM); **10.** *M. polargenta* **sp. n.**, ♂, paratype, northern Vietnam, Cha-pa (MWM). High quality figures are available online.

The Asian members of the genus are middle-sized limacodids with yellowish brown ground colour. The male antennae are broadly bipectinate in basal part. The labial palps are somewhat upcurved, with a very small 3rd segment. The thorax is usually yellow or pale brown. The forewings have diagnostic silver pattern including S-shaped post-median fascia, terminal fascia and a medial silver spot; some of these silver patterns are absent in some species (Figures 1–10). The forewing ground color is paler below the cell. In the forewing the vein R1 is slightly curved and close to Sc; the veins R_3_+R_4_ are branched from R_5_; the medial stem is not divided. The hind tibia has only one pair of spurs, a condition found in other Limacodidae, although two pair also commonly occurs.

**Figures 11–19.**
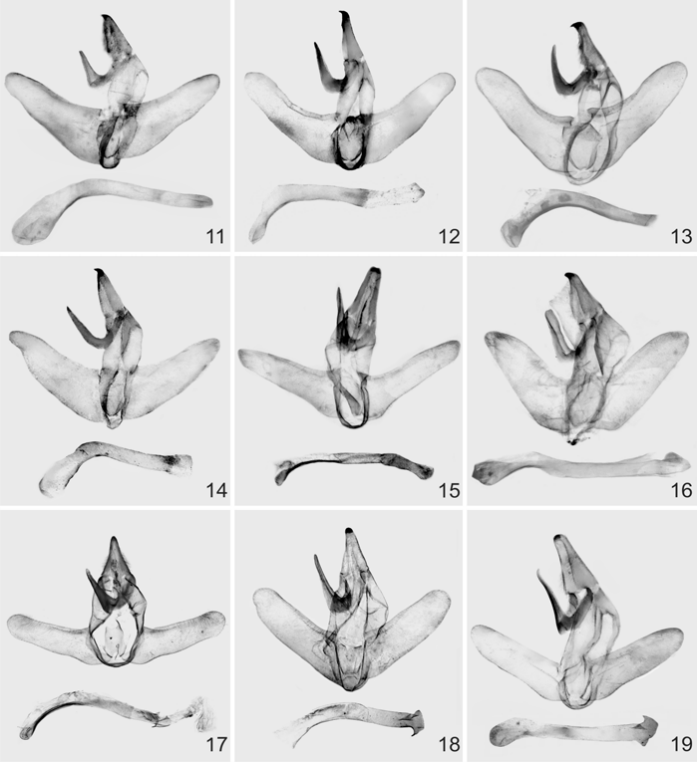
Male genitalia of *Miresa* spp. **11.** *M. bracteata* Butler, China, Xizang, genital slide L05220 (IZCAS); **12.** *M. burmensis* Hering, China, Yunnan, genital slide L05222 (IZCAS); **13.** *M. demangei* de Joannis, genital slide 0113 (CSAV); **14.** *M. fulgida* Wileman, China, Guangdong, genital slide L09001 (IZCAS); **15.** *M. kwangtungensis* Hering, lectotype, China, Guangdong (ZMHB); **16.** *M. urga* Hering, lectotype, China, Siao-Lou (ZMHB); **17.** *M. argentifera* Walker, ♂, holotype, Ceylon (NHM); **18.** *M. fangae* **sp. n.**, paratype, China, Hunan, genital slide L05209 (IZCAS); **19.** *M. polargenta* **sp. n.**, holotype, China, Yunnan, genital slide L05207 (IZCAS). High quality figures are available online.

The male genitalia are not strongly modified (Figures 11–19). The uncus is not divided, usually bears strongly sclerotized apical spur or strong and well defined apical sclerotization. The gnathos is single and strong. The valvae are elongated, without saccular processes. The juxta is simple and flattened. The saccus is short. The aedeagus is long, much longer of the valva, often curved with defined coecum aedeagus basales, and has an apical spur. The vesica is without cornuti.

**Figures 20–25.**
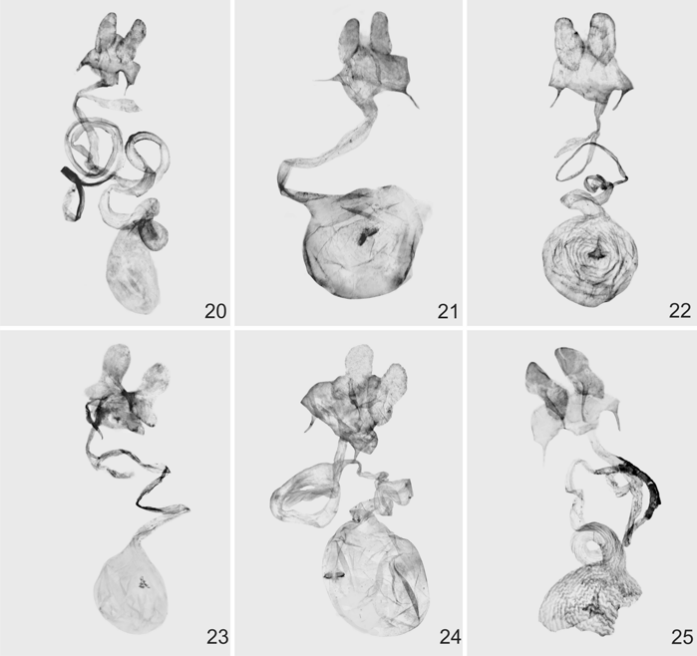
Female genitalia of *Miresa* spp. **20.** *M. bracteata* Butler, China, Xizang, genital slide L05221; **21.** *M. burmensis* Hering, China, Yunnan, genital slide L0S223; **22.** *M. demangei* de Joannis, China, Yunnan, genital slide L05216; **23.** *M. kwangtungensis* Hering, China, Guangxi, genital slide L05211; **24.** *M. urga* Hering, China, Shaanxi, genital slide L05214; **25.** *M. polargenta* **sp. n.**, Paratype, China, Yunnan, genital slide L05208 (all from IZCAS). High quality figures are available online.

In the female genitalia the ductus bursae is spiral; the corpus bursae is ovoid, and bears paired signa (Figures 20–25), the signum can be absent in some species (Figure 20).

The larvae and their host plants are known only for 2 Chinese species. The larvae are of the nettle-type with long scoli; the dorsal scoli on segments A2–A6 are reduced usually. *Coffea* (coffee), *Theobroma, Mangifera* (mango), *Buchanania, Alseodaphne, Terminalia, Aleurites, Canarium* (olive), *Cinchona, Vernicia, Eugenia*, and *Manilkara* are known genera of hosts (Piepers, Snellen 1900; Hering 1931; Holloway 1986; Robinson et al. 2001, 2007).

The genus can be confused with *Narosoideus* Matsumura, 1911, but is well distinguished by the presence of a silver spot or fascia in the forewing. Most probably, the genera *Miresa* Walker and *Narosoideus* Matsumura are closely related and present a monophyletic lineage. Moreover, both genera are probable synonyms distinguished only by presence or absence of any silver pattern on the forewings (Holloway 1986: 88). Both, *Miresa* and *Narosoideus*, have yellowish brown ground color with pale thorax. Their forewings have a similar pattern, almost monotonous ochre-brown, with distinct pale area below the discal cell and usually with a well defined S-shaped post-median fascia which is often silver in *Miresa* and dark in *Narosoideus* (Solovyev and Witt 2009: 106). The male and female genitalia are weakly modified.

**Key to the species in China**
1. Forewing with a triangular silvery spot inside of post-median fascia 2
— Forewing without a triangular silvery spot inside of post-median fascia 5
2. Forewing with a triangular silvery spot in cell 3
— Forewing without a triangular silvery spot in cell 4
3. Forewing with a smaller medial silver spot with width: 1/5 of forewing width and length 1/5.3 of forewing, divided proximally (Figure 5) *M. fulgida*

— Forewing with a larger medial silver spot with width: 1/3.2 of forewing width and length 1/4 of forewing, not divided proximally (Figure 4) *M. demangei*

4. Forewing with a waved post-median fascia, terminal fascia entire (Figures 1, 2) *M. bracteata*

— Forewing with an arched post-median fascia, terminal fascia incomplete (Figure 3) *M. burmensis*

5. Forewing with silvery line indistinctive, partly visible (Figure 6) *M. kwangtungensis*

— Forewing with silvery line distinctive 6
6. Forewing with post-median fascia close to apex at costal margin, almost straight (Figure 7) *M. urga*

— Forewing with post-median fascia far from apex at costal margin, curved 7
7. Forewing with silvery veins proximally of post-median fascia (Figure 10) *M. polargenta*

— Forewing with veins not silvery before postmedian fascia (Figure 9) *M. fangae*

**Figures 26–33.**
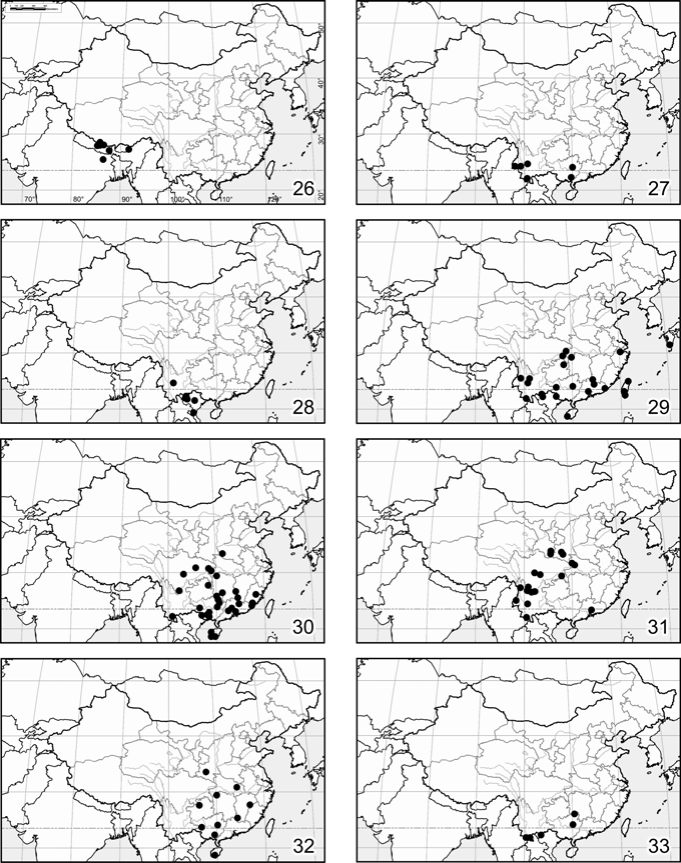
Distribution of *Miresa* spp. **26.** *M. bracteata* Butler; **27.** *M. burmensis* Hering; **28.** *M. demangei* de Joannis; **29.** *M. fulgida* Wileman; **30.** *M. kwangtungensis* Hering; **31.** *M. urga* Hering; **32.** *M. fangae* **sp. n.**; **33.** *M. polargenta* **sp. n**. High quality figures are available online.

***Miresa bracteata* Butler, 1880  (Figures 1, 2, 11, 20, 26)**
*Miresa bracteata* Butler, 1880, *Annals and Magazine of Natural History* (5) **6**: 64. Type locality: [Darjeeling, India]. Lectotype: ♂ (NHM), **here designated**.
***References*:** Cai 1982: 32, 1987: 253 (China: Xizang).
***Diagnosis*:** The species is similar to *fulgida* Wileman, 1910 externally, but defined by the absence of the veins proximal to the silver spot extend silver among them and by the absence of the proximal silver spot in *bracteata* (Figures 1, 2).
***Specimens examined*: China. Xizang:** 2♂♂2♀♀, Zhangmu (2200–2400 m), 25.VI.3.VII.1975, leg. Wang Ziqing & Huang Fusheng; 1♂1♀, Zhangmu, 20.VII.1984, leg. Hu Changsheng (genital slides L05220 (♂), L05221 (♀)); 1♂, Jilong (2800 m), 25.VII.1975, leg. Wang Ziqing (all from IZCAS).
***Distribution*** (Figure 26): Southwestern China (Xizang); India, Nepal, Thailand, Malaysia, Sundaland.
***Biology*:** The flight period is from June and July in China.
***Larva*:** Green with thin yellow wavy lines longitudinally connecting of the abdominal dorsal scoli and with lineation of green and yellow just above subdorsal scoli (Holloway 1986: 89; homology sensu, Epstein 1996). Piepers, Snellen (1900: 73) gave following food plants in Java for misidentified *M. argentifera* Moore: *Cinchona succirubra* Pav., *Coffea arabica* L. (Rubiaceae). Holloway (1986: 89) cited Bell and noted food plants for Indian specimens: *Mangifera* sp., *Buchanania* sp. (Anacardiaceae), *Alseodaphne* sp. (Lauraceae), *Terminalia* sp. (Combretaceae). Robinson et al. (2001, 2007) added following food plants: *Aleurites* sp., *Vernicia montana* Lour. (West Malaysia, Euphorbiaceae), *Coffea liberica* W. Bull ex Hiern (West Malaysia, Rubiaceae), *Eugenia aquea* Burm. f. (West Malaysia, Myrtaceae), *Theobroma cacao* L. (Sterculiaceae), *Manilkara zapota* (L.) P. Royen (India, Sapotaceae).
***Nomenclatorial notes*:** The species *Miresa bracteata* Butler, 1880 was described on the specimens of each sex kept in NHM. The lectotype: male, with following labels: 1 — rounded with red frame and printed text “Type”; 2 — rectangle, yellowish, handwritten by brown (faded black) ink text “*Miresa \ bracteata* ♂ | Butler Type” and in other side “Darjiling | 79·57”; 3 — rectangle, blue, with black printed text “genitalia slide | No.” and black inked “194♂” (Figure 2). This male is supplied with additional lectotype label with corresponding text. The other syntypical specimen, female, is designated as paralectotype.

***Miresa burmensis*Hering, 1931 (Figures 3, 12, 21, 27) New record to China**
*Miresa burmensis* Hering, 1931, in Seitz, *Gross-Schmett. Erde* **10**: 682, Figure 90b. Type locality: “Nieder-Burma” [lower Myanmar]. Holotype: ♂ (NHM).
***Diagnosis*:** The species is distinguishable from other Chinese congeners by having almost uniform dark brown forewings with an ochreous post-median fascia and with a compact silver medial spot; the external and post-median silver fasciae are absent. The species is similar by the forewing pattern to the other Asian members of the genus, *M. albipuncta* (Herrich-Schäffer, 1853); *M. pyronota* Hampson, 1910; and *M. sibinoides* Hering, 1931 known from India and Sri Lanka, but differs in having the aedeagus curvation more than 45° (Figure 12). This diagnostic feature should be verified using more materials from China and the Indian region.
***Specimens examined*: China. Yunnan:** 5♂♂2♀♀, Xiaomengyang (850 m), 24.VIII— 5.IX.1958, leg. Meng Xuwu, Wang Shuyong & Zheng Leyi (genital slides L05222 (♂), L05223 (♀)); 5♂♂, Jinghong (650 m), 21– 24.V.1962, leg. Song Shimei; 3♂♂, Xiaomenglun, 4–6.V.1980, leg. Wang Linyao & Gao Ping; 4♂♂, Xishuangbanna (700 m), 14.IX.1993, leg. Cheng Xinyue; 1♂, Jinghong (650 m), 10.V.1980; 2♂♂, Ruili, Wanding (820 m), 3–5.VI.1979, leg. Zhou Baozhong & Wang Zhijun; 1♀, Jingdong, Dongjiafen (1250 m), 16.VI.1956, leg. Zagulyaev. **Guangxi:** 1♂, Bobai (60 m), 4.X.1983 (genital slide L05228); 1♂, Jinxiu (900 m), 18.V.1999 (genital slide L05224) (all from IZCAS).
***Distribution*** (Figure 27): Southern China (Yunnan, Guangxi); Myanmar, southern Vietnam.
***Biology*:** The specimens were collected in May, June, and August — October at the altitudes of 60–1250 m in China.

***Miresa demangei* de Joannis, 1930 (Figures 4, 13, 22, 28)**
*Miresa demangei* de Joannis, 1930, *Annales de la Société Entomologique de France* **98**: 574. Type locality: “Cha pa” [northern Vietnam, Lao Cai]. Holotype: ♂ (MNHN).
***References*:** Hering 1931: 682 (southern China; as *Miresa fulgida demangei* de Joannis); Luh 1946: 73 (southern China).
***Diagnosis*:** The species is defined from other congeners by a very large medial silver spot with length of 1/3.2 forewing, without proximal silver veins, with concave forewing costa in male (Figure 4). See *fulgida* below.
***Specimens examined*:** 4♂♂1♀, **China, Yunnan**, Jinping (1700 m), 11–15.V.1956, leg. Huang Keren (IZCAS, genital slides L05215(♂), L05216(♀)).
***Distribution*** (Figure 28): Southwestern China (Yunnan); Vietnam.
***Biology*:** The specimens were collected in mid May on the altitude of 1700 m in China.

***Miresa fulgida* Wileman, 1910  (Figures 5, 14, 29)**
*Miresa fulgida* Wileman, 1910, *Entomol*. **43**: 192. Type locality: “Kanshirei (1000 ft.)” [Taiwan]. Lectotype: ♂ (NHM), **here designated**.
*Miresa bracteata* var. *orientis* Strand, 1915, *Suppl. Ent*. **4**: 6. Type locality: “Karapin (Japan)” [Taiwan]. Lectotype: ♂ (RMNH), **here designated**.
*Miresa bracteata* ab. *kagoshimensis* Strand, 1915, *Suppl. Ent*. **4**: 7. Type locality: “Kagoshima (Japan)”. Holotype: ♂ (RMNH).
***References*:** Hering 1931: 682 (Taiwan); Strand 1925: 45 (as *M. bracteata* var. *orientis* Strand and *M. fulgida* Wileman, Taiwan); Hering 1931 (southern China, Taiwan); Matsumura 1931: 115 (Taiwan); Luh 1946: 73 (Formosa, South China); Chang 1989: 176 (Taiwan); Inoue 1992: 101 (Taiwan); Wang 1995: 81 (Taiwan).
***Diagnosis*:** The species is similar to *demangei* de Joannis and *bracteata* Butler, but its silver medial spot in forewing is smaller, with additional veins proximal to the silver spot extend the silver along them and additional proximal silver spot (Figure 5).
***Specimens examined*: China, Yunnan:** 4♂♂, Menghai (1200 m), 18–20.VII.1958, leg. Wang Shuyong; 3♀♀2♂♂, the same, but 11– 17.VII.1978, leg. Luo Hengwen; 2♂♂, Hekou (80–100 m), 5–6.VI.1956, leg. Huang Keren; 2♂♂1♀, Jingdong (1170 m), 1.VI.1956, leg. Zagulyaev; 6♂♂, Pingbian (1500 m), 17– 21.VI.1956, leg. Huang Keren; 1♂, Yongping (1100 m), 15.VI.1980; 1♂, Longchuan (1000 m), 21.VI.1978. **Fujian**: 1♂, Mt. Wuyi (740 m), 14.VI.1960, leg. Zuo Yong; 5♂♂, the same, data but 5.VI.1982, leg. Song Shimei & Zhang Baolin; 2♂♂, the same data, but 15– 18.IX.1982, leg. Zhang Baolin; 13♂♂1♀, the same data, but 11.V-14.VI.1983, leg. Zhang Baolin & Song Shimei; 1♂, Jiangle, Mt. Longqi (900 m), 12.VIII.1991, leg. Song Shimei. **Jiangxi**: 1♂, Dayu (550 m), 14.VI.1977, leg. Liu Youqiao; 1♂, the same data, but 10.VIII.1985, leg. Wang Ziqing; 1♂, Mt. Jiulian, 25.VII.1975, leg. Song Shimei. **Zhejiang**: 1♂1♀, Hangzhou, 9.VI.1976, leg. Chen Ruijing. **Hunan**: 1♂, Sangzhi (1300 m), 11.VIII.1988, leg. Chen Yixin. **Chongqing**: 1♂, Pengshui (750 m), 9.VII.1989, leg. Yang Longlong. **Hubei**: 3♂♂, Lichuan (800 m), 22–23.VII.1989, leg. Li Wei. **Hainan**: 8♂♂, Jianfengling (700–900 m), 18.III-13.IV. 1980, leg. Zhang Baolin & Cai Rongquan; 1♀, the same data, but 11.VIII.1982, leg. Chen Qingzhi; 2♂♂, the same data, but 7– 8.VI.1973, leg. Cai Rongquan. **Guangxi**: 18♂♂2♀♀, Napo (1350 m), 15–19VI.2000, leg. Li Wenzhu & Yao Jian (genital slide L05217); 4♂♂1♀, Jinxiu (900 m), 17– 19.V.1999, leg. Zhang Xuezhong & Li Wenzhu (genital slide L05218); 2♂♂, Jinxiu (600 m), 20.V.1999, leg. Zhang Xuezhong; 4♂♂2♀♀, Jinxiu (400 m), 15.V.1999, leg. Liu Dajun; 1♂, Fangcheng (200 m), 26.V.1999, leg. Zhang Xuezhong; 1♂, Pingxiang, 17.VI.1976, leg. Zhang Baolin. **Guangdong**: 3♂♂, Guangzhou, 20.VIII- 9.IX.1958, leg. Wang Linyao; 5♂♂, the same data, but 16–20.IV.1978, leg. Bai Jiuwei; 2♂♂1♀, VI.1982,leg. Xie Zhenglun (all from IZCAS). **Taiwan**: 1♂, Kosempo, 7.VII.1911 (paralectotype of *M. bracteata* var. *orientis* Strand, 1915); 1♂, the same data, but 12.VII.1911; 1♂, the same data, but 22.VIII.1911; 1♂, the same data, but X.1911; 1♂, “Shis 5 6”, V–VI. 1912 (paralectotype of *M. bracteata* var. *orientis* Strand, 1915); 1♂, Sokutsu, Banshoryo Distr.
***Distribution*** (Figure 29): China (Zhejiang, Jiangxi, Fujian, Guangdong, Guangxi, Hainan, Hubei, Hunan, Sichuan, Yunnan, Taiwan); southern Japan, Vietnam.
***Biology*:** The flight period falls on March and mid May—September in China. The habitat altitudes are 80–1350 m.
***Larva*:** It is of the nettle-type, with four rows of scoli. The mature larva has long dorsal scoli present on segments T3, A1, and A7 only; subdorsal scoli of segments T2, T3, and A2–A9 are short, well developed. The larva is green with pair of dorsal, waved, edged by dark green, white fasciae, running from A1 to A7 where they are joined together; with white, edged by dark green, dorsal, ovoid rings between T2 and T3, T3 and A1. The food plants: *Camellia* spp., *Canarium album* (Lour.) Rausch. (Burseraceae) (Hering 1931: 682; Robinson et al. 2001, 2007). There is one generation per year in Xishuangbana Prefecture, Yunnan Province. It overwinters as the mature larva in the cocoon. The larva feeds during late June to October.
***Remarks*:** The type locality of the lectotype of *Miresa bracteata* var. *orientis* Strand, 1915 is “Karapin (Japan)”. Really, the locality “Karapin” is situated not in Japan. Another name of the locality is “Chaoliping”; it is a village near Fenchihu, Chiayi (Owada 1994: 93).
***Nomenclatorial notes*:** The species *Miresa fulgida* Wileman, 1910 was described based on two specimens of both sexes kept in NHM. The lectotype is male, here designated, with following labels: 1 — rounded with red frame, and black printed text “Type”; 2 — rectangle, with black printed text “Kanshirei, | Formosa. | 1,000 ft. | A.E. Wileman”; handwritten by black ink “24.IV.1908” and red inked “♂”; 3 — rectangle, yellowish with printed black text “Wileman Coll. | B.M. 1929–261.”; 4 — rectangle, yellowish with handwritten by A.E. Wileman, black inked text “*Miresa fulgida* | Type ♂ sp. n.”; 5 — rectangle, yellow, with red inked text “666 T+”. The lectotype is supplied by additional label with corresponding text. The rest syntypical female is designated as paralectotype.
  *Miresa bracteata* var. *orientis* Strand, 1915 was described on 16 male syntypes from “Kosempo” [Taiwan], “Shis A 5 6” [Taiwan], “Sokutsu, Banshoryo-Distr.”, “Karapin (Japan)”. Only single male from Karapin was found in RMNH. The locality “Karapin” is only known in Taiwan, Chiayi County, not in Japan as it was noted in original description and type-label. It conforms to distribution of *Miresa fulgida* Wileman and localities of the rest males in type series of *orientis* Strand. The lectotype of *bracteata* var. *orientis* Strand, 1915 is male, here designated, with following labels: 1 — rectangle, red, with black printed text “Syntypus”; 2 — rectangle, yellowish, with handwritten by E. Strand by black ink text “*Miresa brae*- | *teata* Btl. | ♂ | v. *orientis* m.” and black printed “Strand det”; 3 — rectangle, yellowish, with black printed text “Karapin, VIII.11. | Japan, H. Sauter”. The lectotype is supplied with additional label with corresponding text. The rest syntypical males are designated as paralectotype.

***Miresa kwangtungensis*Hering, 1931, stat. n.  (Figures 6, 15, 23, 30)**
*Miresa argentifera kwangtungensis* Hering, 1931, in Seitz, *Gross-Schmett. Erde* **10**: 683. Type locality: “Kwang-tung, Tsha-yün-schan” [China, Guangdong]. Lectotype: ♂ (ZMHB), **here designated**.
***References*:** Luh 1946: 73 (Tshayunshan, Lofaoshan, Kwangtung; as *M. argentifera kwangtungensis* Hering); Leech 1899: 104 (Central China; as *Miresa decedens* Walker, 1855)
***Diagnosis*:** The species is well recognized from other congeners by uniform dark forewing coloration and deep yellow thorax (Figure 6). Silver medial spot and post-median fascia are absent; the silver terminal fascia is indistinct. In male genitalia the narrower valva and uncus with two apical strongly sclerotized lobes are diagnostic (Figure 15).
***Specimens examined*: China. Guangxi**: 2♂♂, Jinxiu (600 m), 11–17.V.1999, leg. Li Wenzhu & Liu Dajun; 1♂, the same data, but 29.VI.2000, leg. Yao Jian; 3♂♂3♀♀, Jinxiu (200–400 m), 15–16.V.1999, leg. Han Hongxiang & Huang Fusheng (genital slides L05210 (♂), 05211 (♀)); 4♂♂, Jinxiu (1100 m), 10–12.V. 1999, leg. Li Wenzhu & Han Hongxiang; 3♂♂, Jinxiu (1100 m), 10.V.1999, leg. Liu Dajun & Li Wenzhu; 1♂, Shangsi (300 m), 27.V.1999, leg. Yuan Decheng; 1♂, Napo (1440 m), 3.IV.1998, leg. Li Wenzhu; 1♀, Napo (550 m), 22.VI.2000, leg. Zhu Chaodong; 6♂♂, Fangcheng (200 m), 24–26.V.1999, leg. Li Wenzhu; 1♀, Longsheng, 10.VI.1980, leg. Wang Linyao; 2♂♂1♀, Guilin (200 m), 12–14.V.1963, leg. Wang Chunguang; 6♂♂, the same data, but 10–12.IX.1953; 2♂♂, the same data, but 16.VI.1976; 1♂, the same data, but 17.VII.1979; 2♂♂, Guilin, 7–8.VI.1980; 1♂, Qinzhou, 12.IX.1979; 1♂, Longzhou (330 m), 15.VI.2000, leg. Li Wenzhu; 2♀♀, Nanning, 7.VI.1973; 1♀, Yangshuo (160 m), 22.VII.1963, leg. Wang Chunguang; 1♂1♀, Longsheng, 11–13.VI.1980, leg. Wang Linyao & Song Shimei. **Fujian**: 6♂♂, Nanjing, 24– 27.VII.1973, leg. Chen Yixin; 1♂, Jiangle, Mt. Longqi (900 m), 8.VIII.1991, leg. Song Shimei; 1♂, Youxi, 7.V.1976, leg. Huang Bangkan; 1♀, Mt. Wuyi, 12.VIII.1979, leg. Song Shimei. **Hubei**: 1♂, Lichuan (800 m), 23.VII.1989, leg. Li Wei. **Yunnan**: 6♂♂, Xishuangbanna (700 m), 14–15.IX.1993, leg. Yang Longlong. **Sichuan**: 2♂♂1♀, Mt. Emei, 13–19.VI. 1957, leg. Zhu Fuxing & Huang Keren; 2♂♂, the same data, but VII. 1977, leg. Wang Ziqing (genital slide L05212); 5♂♂, 6– 22.V.1979, leg. Wang Linyao & Bai Jiuwei; 1♀, Nanchong, 6.VII.1973; 1♂, Huili, 22.VII.1974, leg. Han Yinheng. **Chongqing**: 1♂, Wanzhou (1200 m), 12.VIII.1993, leg. Song Shimei. **Hunan**: 1♂, Dong'an, 10.VI.1969; 1♂, Sangzhi (1300 m), 11.VIII.1988, leg. Li Wei. **Guangdong**: 1♂, “Kwang-tung, Tsha-yün-schan (lectotype of *M. argentifera kwangtungensis* Hering, 1931; ZMHB); 1♀, “Kwang-tung, Lo-fao-shan” (paralectotype of *M. argentifera kwangtungensis* Hering, 1931; ZMHB); 1♂, Mt. Dinghu, 2.VI.1978, leg. Zhang Baolin; 1♀, the same data, but 11.VI.1973. **Hainan**: 1♂, Jianfengling, 8.VII.1982, leg. Liu Yuanfu; 4♂♂, Baoting, 24.V.1973, leg. Cai Rongquan. **Jiangxi**: 2♂♂, Mt. Jiulian, 11.VI– 28.VII.1975, leg. Song Shimei; 1♂, the same data, but 21.V.1977; 2♂♂, Dayu, 14– 23.VIII.1985, leg. Wang Ziqing; 3♂♂, Dayu (550 m), 17.VI.1977, leg. Liu Youqiao; 1♂, Jinggangshan, 2.VII.1975, leg. *Zhang* Baolin. **Henan**: 1♂, Shangcheng (700 m), 12.VII.1999, leg. Shen Xiaocheng; 1♂, Xixia, 18.VII.1998 (all previous specimens are from IZCAS, if specially not indicated).
***Distribution*** (Figure 30): China (Guangxi, Fujian, Hubei, Yunnan, Sichuan, Hunan, Guangdong, Hainan, Jiangxi, Henan); northern and central Vietnam.
***Biology*:** The specimens were collected in April, May, June, and July on the altitudes of 160–1440 m in China.
***Remarks*:** The taxon *Miresa argentifera kwangtungensis* Hering, 1931 is raised to full specific status because of its strong morphological differences with nominate subspecies *Miresa argentifera argentifera*. The taxon *kwangtungensis* is much darker than *argentifera*. The forewing pattern is rather different with a single not well defined terminal silver fascia in *kwangtungensis* and with both, terminal, and post-median, fasciae in *argentifera*. The male genitalia of *kwangtungensis* differ considerably from *argentifera* and from other members of *Miresa*, and are characterized by the unique for *Miresa* morphology of uncus; it has two apical strongly sclerotized lobes. So, the phylogenetic relationships between both, *kwangtungensis* and *argentifera*, are not proved and the taxon *kwangtungensis* is regarded as a separate species.
  Perhaps, the species *kwangtungensis* was misidentified as *Miresa decedens* Walker, 1855 by Leech (1899: 104) because of external similarity of both species. *Miresa decedens* ranges to the Indian region only.
***Nomenclatorial notes*:** The species was described on two specimens from “Tsha-yünschan” (male) and “Lo-fao-shan” (female); both syntypes were examined in ZMHB. The lectotype is male, here designated, with the following labels: 1 — rectangle, red, with black printed text “Typus”; 2 —- rectangle, yellowish, with printed text “det. Mart. Hering” and black inked by hand of M. Hering “*Miresa Kwang*- | *tungensis* m. | ♂-Type”; 3 — rectangle, yellowish, handwritten by pencil “U 1882”; 4 — rectangle, with printed black text “140528”. The rest syntypical female designated as a paralectotype and supplied with additional lectotype labels.

***Miresa urga*Hering, 1933  (Figures 7, 16, 24, 31)**
*Miresa urga* Hering, 1933, in Seitz, *Gross-Schmett. Erde*, Suppl. **2**: 206, 15 d. Type locality: “Siao-Lou” [China, Sichuan]. Lectotype: ♂ (ZMHB), **here designated**.
***References*:** Luh 1946: 74 (Siaolu); Fang 1997: 1093 (China: Hubei).
***Diagnosis*:** The species is similar externally to *M. fangae* Wu & Solovyev, sp. n., but the forewings are more elongated with pointed apex (Figure 7); the aedeagus bears the large, apical, dorsal, rounded, crest-shaped process (Figure 16).
***Specimens examined*: China. Hubei**: 2♂♂, Xingshan (1200 m), 18–21.VI.1993, leg. Huang Ruizhi; 3♂♂, Shennongjia, 13.VI.1985. **Gansu**: 4♂♂, Kangxia (1000 m), 10–11.VII.1999, leg. Zhu Chaodong & He Tongli; 5♂♂, Kangxian (1400 m), 7– 8.VII.1999, leg. Wang Hongjian, Zhu Chaodong & Yao Jian; 1♂, Chengxian (1020 m), 4.VII.1999, leg. Yao Jian. **Shaanxi**: 1♂, Foping (1750 m), 28.VI.1999; 1♂1♀, Foping (890 m), 26.VI.1999, leg. Yao Jian (genital slides L05213 (♂), L05214 (♀)); 3♂♂1♀, Ningshan (1580 m), 25.VI-1.VII.1999, leg. Yuan Decheng. **Sichuan**: 1♂1♀, Siao-Lou (ZMHB, lectotype and paralectotype); 1♂, Panzhihua, 13.VI.1981; 2♂♂, 21– 24.VII.1974; 2♂♂, Luding (1920 m), 14.VI.1983, leg. Cai Huaicheng; 1♂, Luding (1600 m), 19.VI.1983, leg. Chen Yuanqing; 1♂, Huili, 24.VII.1974, leg. Han Yinheng; 5♂♂, Mt. Emei (800–1000 m), 7.VI—6.VII.1957, leg. Zhu Fuxing, Wang Zongyuan & Huang Keren; 1♂, the same data, but 12.VI.1974, leg. Wang Ziqing. **Chongqing:** 1♂, Wulong (1400 m), 5.VII.1989, leg. Mai Guoqing. **Yunnan**: 3♂♂, Jinping (1700 m), 10–14.V.1956, leg. Huang Keren; 1♀, Weixi (2370 m), 23.VI.1979; 1♂, Tengchong (1750 m), 25.V.1979; 1♂, Yongsheng (2400 m), 8.VII.1980, leg. Liu Dajun; 1♂, Menghai (1200 m), 18.VII.1958; 1♀, Ninglang (2450 m), 15.VII.1979 (all previous specimens are from IZCAS, if specially not indicated); 1♂, Yunlong, Fengshuining Mts (2460 m), 10– 20.V.1999, leg. R. Brechlin (MWM).
***Distribution*** (Figure 31): China (Hubei, Gansu, Shaanxi, Sichuan, Chongqing, Yunnan); northern Thailand, northern Vietnam.
***Biology*:** The moths were collected in mid May – July on the altitudes of 800–2460 m.
***Nomenclatorial notes*:** The species was described from 2 syntypes (couple) from Siao-Lou. The lectotype: ♂ (ZMHB), here designated, with the following labels: 1 — rectangle, red, black printed “Typus”; 2 — rectangle, whitish, black printed “ex coll. | Oberthür”; 3 — rectangle, yellowish, with black frame and handwritten text “Siao-Lou | 1900 | Chasseurs indigènes”; 4 — rectangle, yellowish, with handwritten by M. Hering black inked text “*Miresa* | *urga* m. | ♂-Type” and black printed “det. Mart. Hering”. The other syntypical female is designated here as a paralectotype and supplied with additional label containing corresponding text.

***Miresa fangae* Wu & Solovyev, sp. n.  (Figures 9, 18, 32)**
***Diagnosis*:** The new species is similar to *Miresa argentifera* Walker (known from Sri Lanka and Nepal) (Figures 8, 17), but distinguished by more obscure coloration dark brown hindwings, and abdomen. In male genitalia the valvae and aedeagus of *fangae*, sp. n. are broader, all three apical processes of the aedeagus are distinctly larger, with not spur-like in distal half ventral process. It differs from the sympatric *polargenta*, sp. n. externally by the post-median silver fascia is indistinct and the veins M_2_ and CuA_2_ are not silvery proximally to post-median fascia in forewing; in male genitalia the uncus are not robust and rounded apically, not S-shaped gnathos in lateral view, curved aedeagus in *fangae*.
***Description*:** The wing expanse is 26–33mm. The head and thorax are yellowish brown; tegulae and metathorax are edged with reddish brown (Figure 9). The male antennae are broadly bipectinate in their basal third. The abdomen is reddish brown. The forewings are dark reddish brown with yellowish brown area below cell; the post-median fascia is waved, and indistinct excepting its inner margin part; the terminal fascia is composed of a row of the silvery dots. The hindwings are reddish brown.
  In the male genitalia the uncus is short and wide; the gnathos is well developed, wide, apically hooked, and gradually slenderized distally; the valvae are narrow and long, slightly tapering to a rounded apex; the juxta is sclerotized weakly, shield-shaped; the aedeagus is slender and long, slightly arched proximally, and contains 3 flattened, compact, triangular spurs, the ventral one is wide, gradually narrowed to the apex (Figure 18).
***Holotype*:** ♂, China, Hainan: Tongshi, 2.VI.1973, leg. Cai Rongquan (genital slide L05206, IZCAS).
***Paratypes*: China. Hainan**: 1♂, Wuzhi-Shan Mts, 18°53′N, 109°43′E (1500 m), 1– 12.IV.2003, leg. Siniaev & his team (MWM, genital slide 14354); 3♂♂, China, Hainan, Wuzhi-Shan Mts., 18° 53′ N, 109° 43′ E, 1500 m, 20.II-10.IV.2001, leg. local collector (MWM). **Jiangxi**: 2♂♂, Dayu, 15.VII.1975, leg. Song Shimei (IZCAS); 1♂, Wuyi Shan, Xipaihe village, 27° 54′ N, 117° 20′ E, 1500 m, VIII.2004, leg. Siniaev & his team (MWM, genital slide 16128). **Hunan**: 1♂, Sangzhi (1300 m), 11.VIII.1988, leg. Chen Yixin (IZCAS, genital slide L05209). **Guangxi**: 2♂♂, Napo (550 m), 22.VI.2000, leg. Li Wenzhu & Zhu Chaodong (IZCAS); 2♂♂, Napo (1350 m), 18.VI.2000, leg. Yao Jian (IZCAS); 1♂, Jinxiu, 22.VIII.1981, leg. Hou Bangquan (IZCAS); 1♂, Liuwan, 20.X.1981, leg. Lai Laian (IZCAS). **Guizhou**: 1♂, Mt. Fanjing (500 m), 11.VII.1988, leg. Li Wei (IZCAS). **Hubei**: 3♂♂, Wuhan City, Tapieh Shan, 900–1600 m, mid VI — late VIII.1999, leg. J. Li (MWM, genital slide 16127). **Shaanxi**: 1♂, Taibaishan Mt., Tsinling Mts, 33° 55′ N, 107° 44′ E, 1900 m, VI.2004, leg. V. Siniaev & his team (MWM, genital slide 16129).
***Distribution*** (Figure 32): China (Jiangxi, Hunan, Guangxi, Hainan, Guizhou, Hubei, Shaanxi), central Vietnam.
***Biology*:** The flying period falls on early April, June–August and mid October. The habitat altitudes are 550–1900 m.
***Etymology*:** The species is named in honour of Prof. Chenglai Fang for her contribution to the classification of the Chinese Limacodidae.

***Miresa polargenta* Wu & Solovyev, sp. n.  (Figures 10, 19, 25, 33)**
***Diagnosis*:** The species is similar to *M. argentifera* Walker and *M. fangae* Wu & Solovyev, sp. n., but differs by the forewing with silvery veins M2 and CuA2 proximally to the silver post-median fascia; darker hindwings. In male genitalia the robust and rounded apically uncus, S-shaped in lateral view gnathos, shorter, almost straight aedeagus bearing apically a characteristic craniad dorsal triangular process and not spur-like (as in *argentifera*) ventral process are diagnostic and well separate *polargenta* from these similar species.
***Description*:** The wing expanse is 26–33 mm. The head and thorax are yellowish brown; the tegulae and metathorax are edged with reddish brown (Figure 10). The abdomen is reddish brown. The forewings are dark reddish brown, but are pale reddish brown below cell; the post-median fascia is waved; the veins M_2_ and CuA_2_ are silvery before post-median fascia; the terminal fascia is composed of a row of silvery dots. The hindwings are reddish brown.
  In male genitalia (Figure 19) the uncus is short and wide, rounded apically; the gnathos is well developed, apically hooked; the valvae are narrow and long, tapering to a rounded apex; the juxta is sclerotized weakly, shield-shaped; the aedeagus is narrow and long, almost straight, apically has 3 triangular processes on the top.
  In female genitalia (Figure 25) the anterior apophyses are as short as 1/3 of the posterior ones; the ductus bursae is very long; the corpus bursae is ovate, large; the paired signa elongated along symmetry axis, strongly widened cranially, bearing spines.
***Holotype*:** ♂, China, Guangxi: Jinxiu (1100 m), 2.VII.2000, leg. Li Wenzhu (IZCAS, genital slide L05205).
***Paratypes*: China. Guangxi**: 1♂, Mt. Miaoer (1150 m), 6.VII.1985, leg. Fang Chenglai (IZCAS); **Yunnan**: 1♂1♀, Menghai (1200 m), 5–10.VIII.1982, leg. Luo Hengwen (IZCAS, genital slides L05207 (♂), 05208 (♀)); 1♂, Menglun, 3.VI.1964, leg. Zhang Baolin (IZCAS). **Vietnam. Lao Cai**: 2♂♂, Fan-si-pan Mts, near Chapa, 22° 20′ N, 103° 40′ E (1600–1800 m), V.1995, leg. local collectors (MWM, genital slide 14353); 1♂, N-side, 22.17° N, 103.44° E (1525 m), 7, 10.VII.1994, leg. Brechlin & Schintmeister (MWM, genital slide 14359); 1♂, W-side, 22.20° N, 103.40° E (1600–1800 m), 30.VI– 12.VII.1994, leg. Brechlin & Schintmeister (MWM); 4♂♂, Sa Pa N.P. (1500 m), 10– 20.V.2006, leg. V. Zolotuhin (CSAV, alcohol preparation VZ-LIMAC 44, 45).
***Distribution*** (Figure 33): Southern China (Guangxi, Yunnan); northern Vietnam (Lao Cai).
***Biology*:** The moths were collected in May, June—mid July and early August on the altitudes of 1100–1800m.
***Etymology*:** The name is derived from Greek “poly” (= numerous) and “argentum” (= silver), corresponding to the numerous silvery stripes on the forewing.

## Conclusions

The Chinese species of the genus *Miresa* Walker, 1855 are reviewed. In total 8 species were found, and 3 of them are newly recorded. Two of these species are described here as new: *M. fangae* Wu & Solovyev, sp. n. (type locality: “Hainan: Tongshi”); *M. polargenta* Wu & Solovyev, sp. n. (type locality: “Guangxi: Jinxiu (1100 m)”). The taxon *M. argentifera kwangtungensis* Hering, 1931 is raised to full specific status. The lectotypes are designated for the following taxa: *M. bracteata* Butler, 1880 (♂, NHM); *M. fulgida* Wileman, 1910 (♂, NHM); *M. bracteata* var. *orientis* Strand, 1915 (♂, RMNH); *M. argentifera kwangtungensis* Hering, 1931 (♂, ZMHB); and *M. urga* Hering, 1933 (♂, ZMHB).

The fauna of the genus in southern China is characterized by hi-level biodiversity, as well as biodiversity of Xizang (Southwestern China) which belongs to Indian fauna; whereas the provinces Yunnan, Guangdong, and Hainan are related to Northern Vietnamese fauna.

The preimaginal stages and larval host plants are known just for 2 species in China in spite of economic importance of the species as pests of different cultural plants. Further investigations of host plants are urgent.

## Abbreviations

**CSAV**,: personal collection of Alexey V. Solovyev;
IZCAS: Institute of Zoology, Chinese Academy of Sciences;
**MWM**,: Museum Witt, Munich, Germany;
**NHM**,: Natural History Museum, London, United Kingdom;
**MNHN**,: Museum National d'Histoire Naturelle Paris;
**RMNH**,: Nationaal Natuurhistorische Museum (“Naturalis”), Leiden, Netherlands (formerly Rijksmuseum van Natuurlijke Historie);
**ZMBH**,: Zoologisches Museum der Humboldt Universität zu Berlin, Germany
